# Evaluation of Kappa Index as a Tool in the Diagnosis of Multiple Sclerosis: Implementation in Routine Screening Procedure

**DOI:** 10.3389/fneur.2021.676527

**Published:** 2021-08-11

**Authors:** Carmen Teresa Sanz Diaz, Silvia de las Heras Flórez, Mercedes Carretero Perez, Miguel Ángel Hernández Pérez, Vicente Martín García

**Affiliations:** ^1^Clinical Analysis Laboratory, Hospital Nuestra Señora de Candelaria, Santa Cruz de Tenerife, Spain; ^2^Neurology Department, Hospital Nuestra Señora de Candelaria, Santa Cruz de Tenerife, Spain; ^3^Radiodiagnosis Department, Hospital Nuestra Señora de Candelaria, Santa Cruz de Tenerife, Spain

**Keywords:** multiple scleorsis, K-index, free light chain, cerebrospinal fluid, oligoclonal bands, biomarkers

## Abstract

Multiple sclerosis (MS) is an inflammatory demyelinating disease of the central nervous system. Previous studies have shown that cerebrospinal fluid (CSF) kappa free light chains (K-FLCs) may have a role in MS diagnosis. In this regard, the kappa index (K-Index) has demonstrated higher sensitivity, and slightly lower specificity than oligoclonal bands (OCBs), the gold standard for the detection of intrathecal immunoglobulin synthesis, a feature of MS. Here, we evaluated the performance of the K-Index (K-Index = CSF/serum K-FLC divided by CSF/serum albumin) for the differential diagnosis of MS in a cohort of patients with suspected MS. K-FLCs were quantitatively measured in parallel serum and CSF samples by turbidimetry (Freelite Mx reagent on an Optilite system, The Binding Site Group Ltd). From 160 (63.4%) of a total of 252 patients who had K-FLC in CSF <0.03 mg/dl, below the sensitivity limit of the technique, only one had a diagnosis of MS. However, the absence of OCB in this same patient suggested no synthesis of intrathecal immunoglobulin. Globally, MS patients presented significantly higher K-Index levels than patients without an MS diagnosis (66.96 vs. 0.025, respectively; *p* < 0.0001). In agreement, patients with positive OCB testing also exhibited higher K-Index levels than patients negative for OCB (65.02 vs. 0.024, respectively; *p* < 0.0001). An optimal K-Index cutoff of 3.045 was defined by receiver operating characteristic (ROC) analysis for screening suspected MS, achieving a higher diagnostic sensitivity and slightly lower specificity than OCB (Sens. 0.9778 and Spec. 0.8629 vs. Sens. 0.8889 and Spec. 0.9086, respectively). A previously reported K-Index cutoff of 6.6 also showed good diagnostic performance (Sens. 0.9333; Spec. 0.8731), validating its power as a diagnostic biomarker for MS. Finally, a time- and cost-effective algorithm for MS screening is proposed that would offer an initial rapid evaluation of the intrathecal immunoglobulin synthesis through the K-FLC in CSF and K-Index analysis, followed by reflexing OCB testing that may be ordered more selectively.

## Introduction

Multiple sclerosis (MS) is an inflammatory demyelinating disease of the central nervous system (CNS), affecting over 2 million persons worldwide. The first symptoms are often displayed as an isolated flare called clinically isolated syndrome (CIS). Most people with MS have relapses followed by a remission period. As the condition progresses, relapses occur more frequently and are followed by shorter recoveries (1). An early diagnosis and treatment are crucial to prevent relapses and the increasing disability of the patient (2, 3).

In MS, when inflammation of the CNS occurs, there usually is a synthesis of intrathecal immunoglobulins (4) that accumulate in the cerebrospinal fluid (CSF), since the blood–CSF barrier (BCB) prevents their leakage into the blood. The gold standard method for detection of those immunoglobulins with high sensitivity (5, 6) is the isoelectric focusing determination of oligoclonal bands (OCBs). Diagnosis criteria for MS, which combine clinical evidences, MRI imaging, data and CSF analysis, are continuously being revised (7). In the latest update, the finding of two or more OCB in CSF was included among the McDonald criteria for the diagnosis of MS, due to its high predictive value for conversion from CIS to MS (7). However, the validation of an alternative method for assessment of intrathecal synthesis of immunoglobulins is of utmost importance, since the OCB isoelectric focusing is expensive, labor-intensive, and highly susceptible to user interpretation.

Several studies have reported an intrathecal synthesis of kappa free light chains (K-FLCs) alongside the synthesis of immunoglobulins in CSF (8–11). The increasing sensitivity of turbidimetric methods makes these techniques suitable to objectively quantify the intrathecal synthesis of immunoglobulins in an automatized way. In fact, the determination of K-FLCs in CSF as a surrogate biomarker for MS has been intensively studied in the past years (6, 12–14). Both parameters, K-FLC in CSF and kappa index (K-Index = CSF/serum K-FLC divided by CSF/serum albumin), have shown to be increased in patients with CIS and MS as compared with control groups (15–17). Additionally, in several studies, a K-Index cutoff has been validated with higher sensitivity and slightly lower specificity than OCB for MS diagnosis (6, 14). These reports support the hypothesis that K-FLC offers a quantitative and easy-to-standardize method for MS diagnosis, and its incorporation as a screening test has been suggested, where it would facilitate the laboratory work and save resources (18, 19).

The purpose of this study was to evaluate the performance of the K-FLC and the K-Index for differential diagnosis of MS in a cohort of patients from our Hospital in Canary Islands. In addition, an optimized diagnosis algorithm for CSF analysis that minimizes the need to perform OCB was established.

## Materials and Methods

### Patients

A total of 276 consecutive and unselected patients, referred to our lab for OCB evaluation, from January 2018 to March 2020, were included. A Multiple Sclerosis Protocol was implemented in 2005 in our center, in consensus with the Neurology Unit (20), which includes the evaluation of the OCB in parallel serum and CSF samples. Patients with insufficient or missing parallel serum or CSF samples were excluded (final *n* = 252). All diagnostics were reviewed by a multidisciplinary group including a neurologist and image and laboratory expert clinicians. Forty-five patients were diagnosed with MS according to the McDonald criteria (7), and the remaining 207 patients included a variety of other diseases of the CNS, such as migraine, optic neuritis, vasculitis, cognitive impairment, autoimmune encephalopathy, cranial hypertension, stroke, meningoencephalitis, motor sensory disorder, Guillain–Barré syndrome, myelitis, and neuromuscular paralysis.

This study was approved by the ethical committee del Hospital Universitario Nuestra Señora de Candelaria translate with the code CHUNSC 2020_110.

### Analytical Determinations

Serum and CSF K-FLC quantitative levels were determined in all patients using the Human Kappa Freelite Mx kit (The Binding Site Group Ltd., Birmingham, UK) on a turbidimetric Optilite system. IgG OCB testing was performed by high-resolution agarose gel electrophoresis and immunofixation with enzyme-labeled antisera on a second-generation semiautomatic Hydrasys Focusing (Sebia Hispania, Barcelona, Spain). A positive OCB was considered if at least two OCBs were visible exclusively in the CSF sample. Serum albumin was measured by colorimetry, and serum IgG and CSF-albumin were measured by immunoturbidimetric spectrophotometry on the Cobas 8000 C-702 and Cobas 6000 C-501 automatic systems (Roche Diagnostics, Basel, Switzerland). The CSF and serum samples were kept frozen at −80°C until the analysis was carried out.

### Statistical Analysis

To account for possible alterations of the blood-brain barrier function, the K-Index was determined in all patients [K-Index = (K-FLC × sALB)/(sFLC-K × CSF-ALB)], aiming at increasing the diagnostic precision. For those patients presenting a K-FLC below the detection limit of the technique (K-FLC < 0.03 mg/dl), an empirical low value of 0.0001 mg/dl, close to zero, was attributed for further statistical analysis. This reflects the eventual clinical practice when patients with undetectable K-FLC levels in CSF are reported as having no evidence of intrathecal immunoglobulin synthesis according to this assay. Patients with CIS, radiologically isolated syndrome (RIS), or an inconclusive final diagnosis were not included in the K-Index's receiver operating characteristic (ROC) curve analysis for MS vs. no MS but were included on the OCB positive vs. negative comparison. Descriptive population analysis, Mann–Whitney, Contingency, and ROC curve analyses were all done on GraphPad Prism, version 8.3.0 software. *p*-Values < 0.05 were considered statistically significant.

## Results

### Intrathecal Immunoglobulin Production

**Figure 1:**
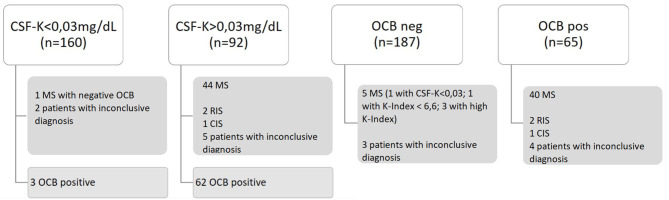
Patient distribution according to detectable (>0.03 mg/dl) or undetectable (<0.03 mg/dl) K-FLC CSF levels and OCB status positive or negative for the presence of >2 oligoclonal bands. MS, multiple sclerosis; OCB, oligoclonal banding; RIS, radiologically isolated syndrome; CIS, clinically isolated syndrome; K-FLC, kappa free light chain; CSF, cerebrospinal fluid.

K-FLC and K-Index identified more MS patients than OCB testing (44 vs. 40). From a total of 252 patients included for intrathecal synthesis evaluation, 160 (63.8%) patients had undetectable K-FLC levels in CSF, of whom 157 did not have MS, two had an inconclusive diagnosis, and one had MS (Figure 1). This MS patient fulfilled clinical and MRI criteria but did not display high K-Index nor positive OCB, and it was therefore, impossible to confirm intrathecal immunoglobulin synthesis. Both patients with an inconclusive diagnosis were also negative for OCB: one patient had transitory visual events compatible with visual aura and left thalamic–mesencephalic lesion with a demyelinating appearance compatible with an inflammatory process, on the process of ruling out atypical variant of MS, Behçet, neuromyelitis optica (NMO), venous vasculitis; and the other patient had ambiguous clinical symptoms, possibly a primary progressive MS, and clear positive MRI results with dissemination in space. Additionally, three patients positive for OCB testing had undetectable K-FLC CSF levels, but none of them had MS. On the other hand, 92 (36.2%) patients had measurable K-FLC CSF levels, of whom 62 were positive and 30 negative for OCB. These 92 cases include 44 MS patients, two cases of RIS, one case of CIS, five cases with and inconclusive diagnosis at the time of the study, and 40 in whom MS was ruled out. Within the 40 cases without MS, 25 presented with a K-Index above the previously published 6.6 cutoff, of whom 13 also had OCB present and tended to have higher K-Index levels, corroborating the intrathecal production of immunoglobulin in those cases.

By OCB testing, 65 (25.8%) out of the 252 patients were positive and corresponded to 40 MS diagnosis, two RIS, one CIS, four patients with inconclusive diagnosis, and 18 cases without MS. Within the 18 cases without MS and positive OCB, 15 had detectable K-FLC CSF levels (>0.03 mg/dl), and three had K-Index levels above 6.6. Of the 187 (74.2%) OCB-negative patients, MS was ruled out in 179, three patients had inconclusive diagnosis, and five patients had MS. All five patients with MS but negative OCB fulfilled MRI criteria for MS. Among them, three presented high K-Index, 1 K-Index <6.6, and one had no evidence of kappa light chain intrathecal synthesis (K-FLC CSF < 0.03 mg/dl).

### Kappa Free Light Chain in Cerebrospinal Fluid and Kappa Index Levels in Multiple Sclerosis Patients

**Figure 2:**
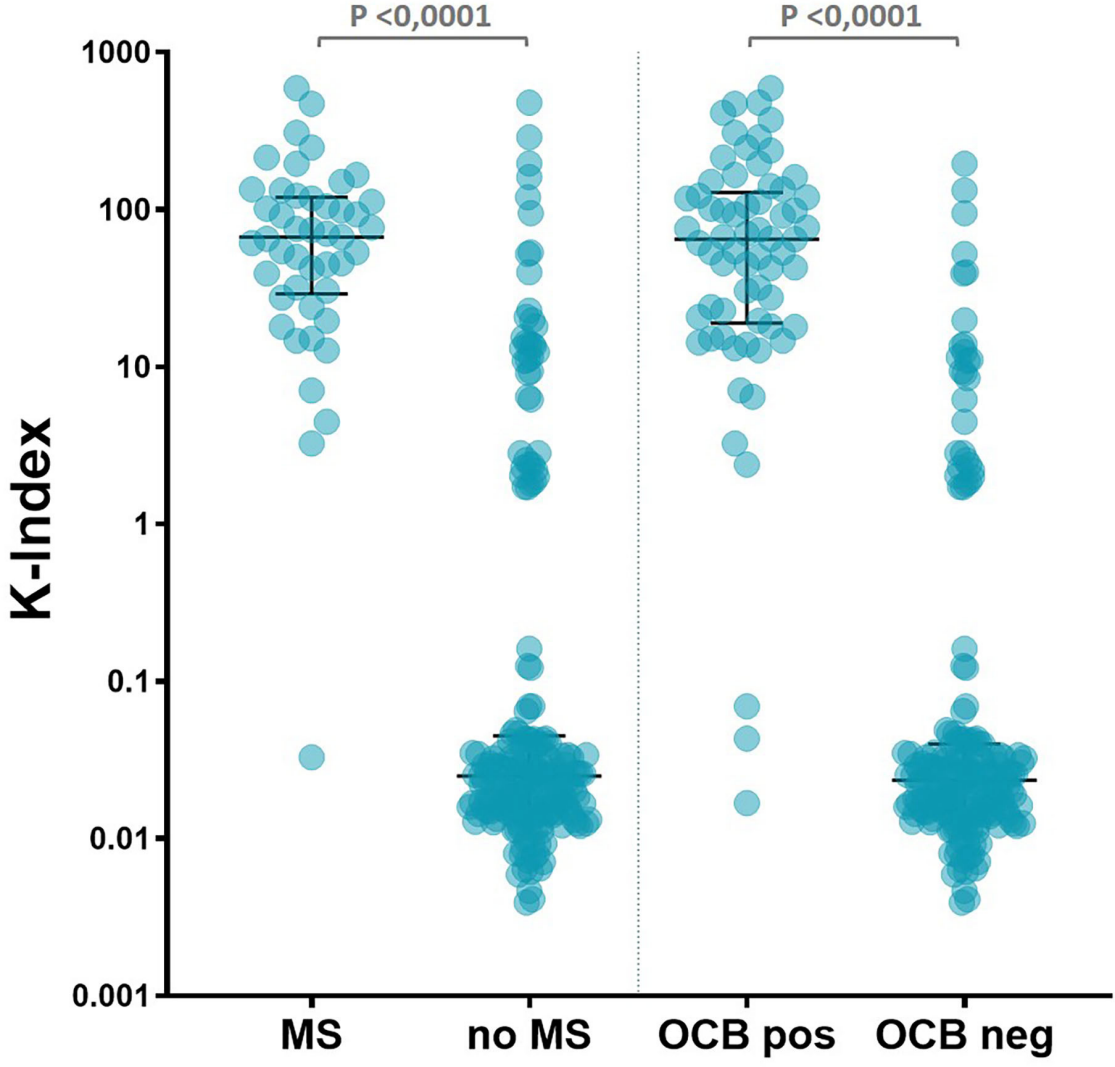
K-Index individual values, group medians, and interquartile range, from patients with MS, without MS, positive OCB, and negative OCB. Significantly different group medians (*p* < 0.0001) by Mann–Whitney test. K-Index, kappa index; MS, multiple sclerosis; OCB, oligoclonal band.

In the present cohort, a total of 45 (17.8%) patients were finally diagnosed with MS (Table 1). Median K-Index levels among MS patients was significantly higher (66.96) than in non-MS patients (0.025) (*p* < 0.0001) (Figure 2 and Table 1). Forty-two out of the 45 (93.3%) MS patients presented a K-Index >6.6, and only three MS patients presented a K-Index below 6.6, a cutoff obtained in the largest multicentric study to date evaluating the K-Index in an MS population (14). Among the three patients with MS and a K-Index <6.6, one was diagnosed with MS based on clinical and MRI evidences, because of undetectable K-FLC levels in CSF and absence of OCB, thereby, showing no evidences of detectable intrathecal synthesis of immunoglobulins. Another patient also with clinical and imaging diagnoses of MS had K-FLC CSF levels of 0.07 mg/dl and a K-Index of 4.48 but was negative for OCB. The third MS patient with K-Index <6.6 (in this case, a K-Index of 3.26, K-FLC CSF of 0.12 mg/dl) corresponds to a patient with positive OCB, diagnosed in 2003 in another clinical center.

**Table 1:** Patient's characteristics.

	**Population**		**Value**
Number of patients	All		252
	MS		45
	CIS		1
	RIS		2
	Inconclusive		7
	Other diagnoses		197
Age (median; years)	All (min.; max.)	*n* = 252	44.12 (6.48; 90.4)
	MS (min.; max.)	*n* = 45	41.39 (16.26; 63.99)
K-FLC in CSF (median; mg/dl)	All (min.; max.)	*n* = 252	0.0001 (0.0001; 1.98)
	**MS** (min.; max.)	*n* = 45	**0.26** (0.0001; 1.98)
	**No MS** (min.; max.)	*n* = 197	**0.0001** (0.0001; 1.64)
	MS/CIS/RIS (min.; max.)	*n* = 48	0.265 (0.0001; 1.98)
	No MS/inconclusive (min.; max.)	*n* = 204	0.0001 (0.0001; 1.64)
	**OCB pos** (min.; max.)	*n* = 65	**0.26** (0.0001; 1.98)
	**OCB neg** (min.; max.)	*n* = 187	**0.0001** (0.0001; 1.64)
K-Index (median)	All (min.; max.)	*n* = 252	0.03134 (0.003912; 593.8)
	**MS** (min.; max.)	*n* = 45	**66.96** (0.03288; 593.8)
	**No MS** (min.; max.)	*n* = 197	**0.02499** (0.003912; 479)
	MS/CIS/RIS (min.; max.)	*n* = 48	66.02 (0.03288; 593.8)
	No MS/inconclusive (min.; max.)	*n* = 204	0.0253 (0.003912; 479)
	**OCB pos** (min.; max.)	*n* = 65	**65.02** (0.01682; 593.8)
	**OCB neg** (min.; max.)	*n* = 187	**0.02351** (0.003912; 195.6)

*Median age, K-FLC in CSF, and K-Index values are obtained for the different population groups. Significantly different K-FLC and K-Index median values were found between MS and no MS groups; MS/CIS/RIS and no MS/inconclusive; and OCB pos and OCB neg groups (Mann–Whitney test, p-value < 0.0001). K-FLC, kappa free light chain; CSF, cerebrospinal fluid; K-Index, kappa index; MS, multiple sclerosis; CIS, clinically isolated syndrome; RIS, radiologically isolated syndrome; OCB, oligoclonal band. Bold values are used to highlight the most important data between different groups*.

### Diagnostic Kappa Index Thresholds for Multiple Sclerosis and/or Oligoclonal Band

**Figure 3:**
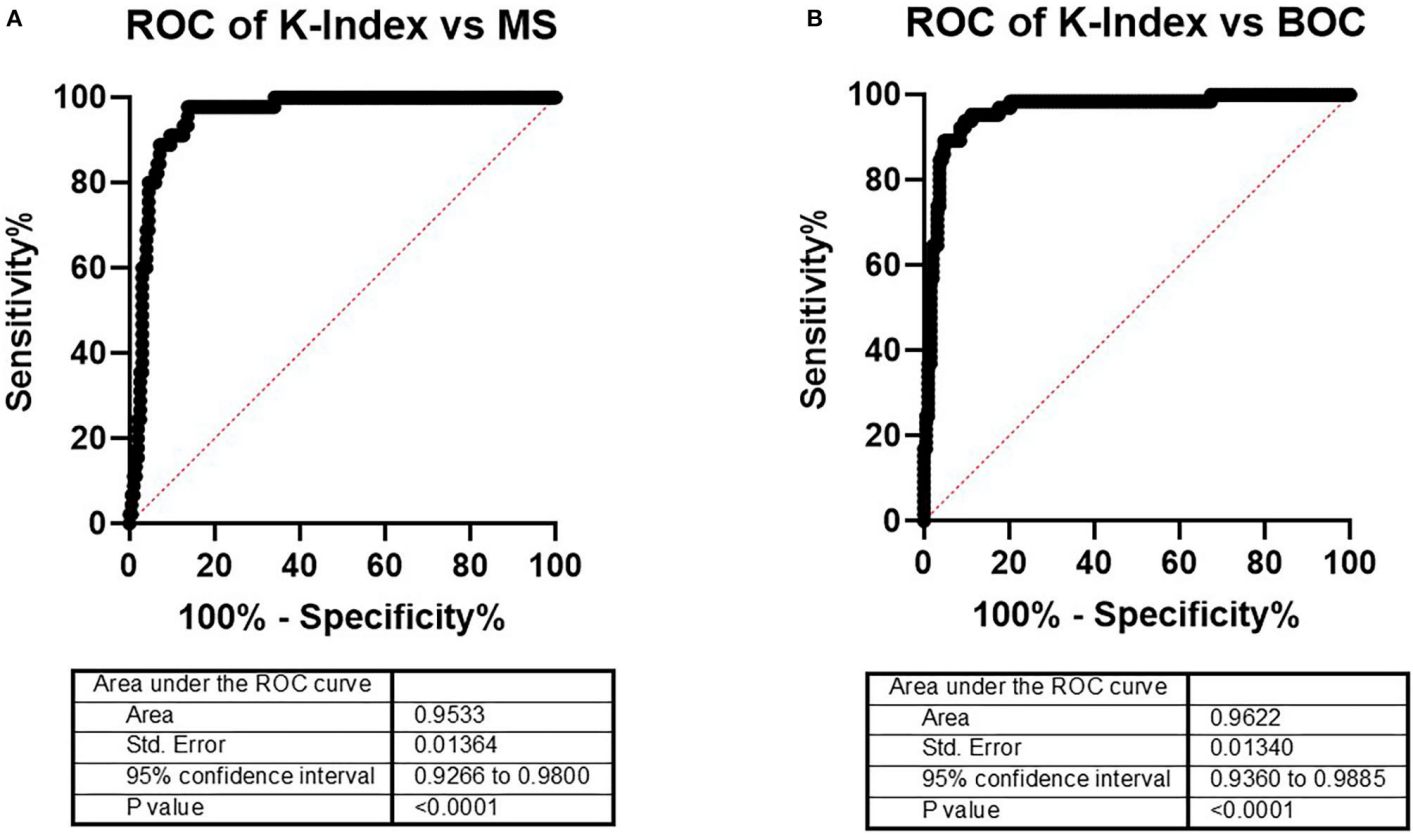
ROC curves for the K-Index performance to discriminate **(A)** MS patients from non-MS patients and **(B)** patients positive from patients negative for OCB. ROC, receiver operating characteristic; K-Index, kappa index; MS, multiple sclerosis; OCB, oligoclonal band.

To improve the diagnostic process, an in-house cutoff was established that favored diagnostic sensitivity. A ROC curve analysis was done with data from both MS diagnosed and MS ruled out patients, excluding isolated syndromes and inconclusive diagnosis. As expected, the performance of the K-Index for MS diagnosis was very good, reflected by an area under the curve (AUC) of 0.9533 (Figure 3). Because the aim of this study was to explore the K-Index as a screening tool for further testing in the differential MS diagnosis process, a ROC curve was also generated to determine its discriminatory power between OCB positive and negative patients. Again, the K-Index showed a significant discriminatory capacity for OCB status, with an AUC slightly higher than that obtained for the MS diagnosis (0.9622). Thus, in both settings, the technique has shown a good diagnostic capacity.

Regarding MS diagnosis, the K-Index threshold that scored best according to the combination of the sensitivity and specificity characteristics from the ROC curve analysis was 3.045 (Table 2). This K-Index cutoff offered a higher sensitivity than the OCB testing for MS (0.9778 vs. 0.8889, respectively) with a slightly inferior specificity (0.8629 vs. 0.9086, respectively). The performance of the previously published 6.6 K-Index cutoff for identifying patients with MS was also confirmed, showing a slightly higher sensitivity and lower specificity when compared with the OCB (Table 2), validating this K-Index cutoff for MS diagnosis in the daily clinical practice.

**Table 2:** Contingency characteristics of OCB testing for MS diagnosis and of different K-Index cutoffs for both MS diagnosis and OCB status.

	**MS diagnosis**				**OCB**		
	**OCB**	**K-Index >3.045**	**K-Index >6.6**	**K-Index >12.58**	**K-Index >3.045**	**K-Index >6.6**	**K-Index >12.58**
**Sensitivity**	**0.8889**	**0.9778**	**0.9333**	**0.9111**	**0.9385**	**0.9077**	**0.8923**
95% CI	0.7650–0.9516	0.8843–0.9989	0.8214–0.9771	0.7927–0.9649	0.8522–0.9758	0.8129–0.9570	0.7940–0.9468
**Specificity**	**0.9086**	**0.8629**	**0.8731**	**0.9036**	**0.9037**	**0.9144**	**0.9519**
95% CI	0.8602–0.9414	0.8079–0.9041	0.8194–0.9125	0.8543–0.9374	0.8530–0.9382	0.8655–0.9466	0.9111–0.9745
**Positive predictive value**	**0.6897**	**0.6197**	**0.6269**	**0.6833**	**0.7722**	**0.7867**	**0.8657**
95% CI	0.5620–0.7938	0.5034–0.7237	0.5072–0.7328	0.5577–0.7869	0.6683–0.8507	0.6812–0.8642	0.7640–0.9277
**Negative predictive value**	**0.9728**	**0.9942**	**0.9829**	**0.978**	**0.9769**	**0.9661**	**0.9622**
95% CI	0.9380–0.9883	0.9676–0.9997	0.9508–0.9953	0.9449–0.9914	0.9421–0.9910	0.9280–0.9844	0.9240–0.9816
**Likelihood ratio**	9.728	7.134	7.355	9.447	9.75	10.61	18.54
***n***	242	242	242	242	252	252	252

*Sensitivity, specificity, and predictive values are presented. OCB, oligoclonal band; MS, multiple sclerosis; K-Index, kappa index. Bold values are used to highlight the most important data between different groups*.

For the identification of OCB positive patients, the best-performance K-Index cutoff identified was of 12.58 (sensitivity 0.8923; specificity 0.9519). However, the 3.045 cutoff had an almost identical performance for OCB status (sensitivity 0.9385; specificity 0.9037) and may be preferred for screening purposes because of its higher sensitivity to identify MS patients as well, regardless of the slightly decreased specificity for OCB results.

Seven out of 252 (2.8%) patients had an inconclusive final diagnosis after laboratory, MRI, and clinical evaluations (Supplementary Table 1). Five of them had unclear MRI results, three of whom presented very high K-Index levels, one with a moderately elevated level (K-Index of 8.49), and one with a very low level, meaning without evidence of intrathecal immunoglobulin synthesis measurable by FLC assay. OCB and K-Index agreed in six clinical cases, with the only exception being the moderately abnormal K-Index of 8.49 from a patient with severe right optic neuropathy under investigation. Follow-up on these patients is required to understand the clinical relevance of the CSF results obtained in the lab. However, to include the K-FLC analysis may be opportune to confirm OCB results when a final diagnosis is difficult to be established.

### Combined Use of Kappa Free Light Chain Cerebrospinal Fluid Levels and Kappa Index Values for Optimized Multiple Sclerosis Screening

**Figure 4:**
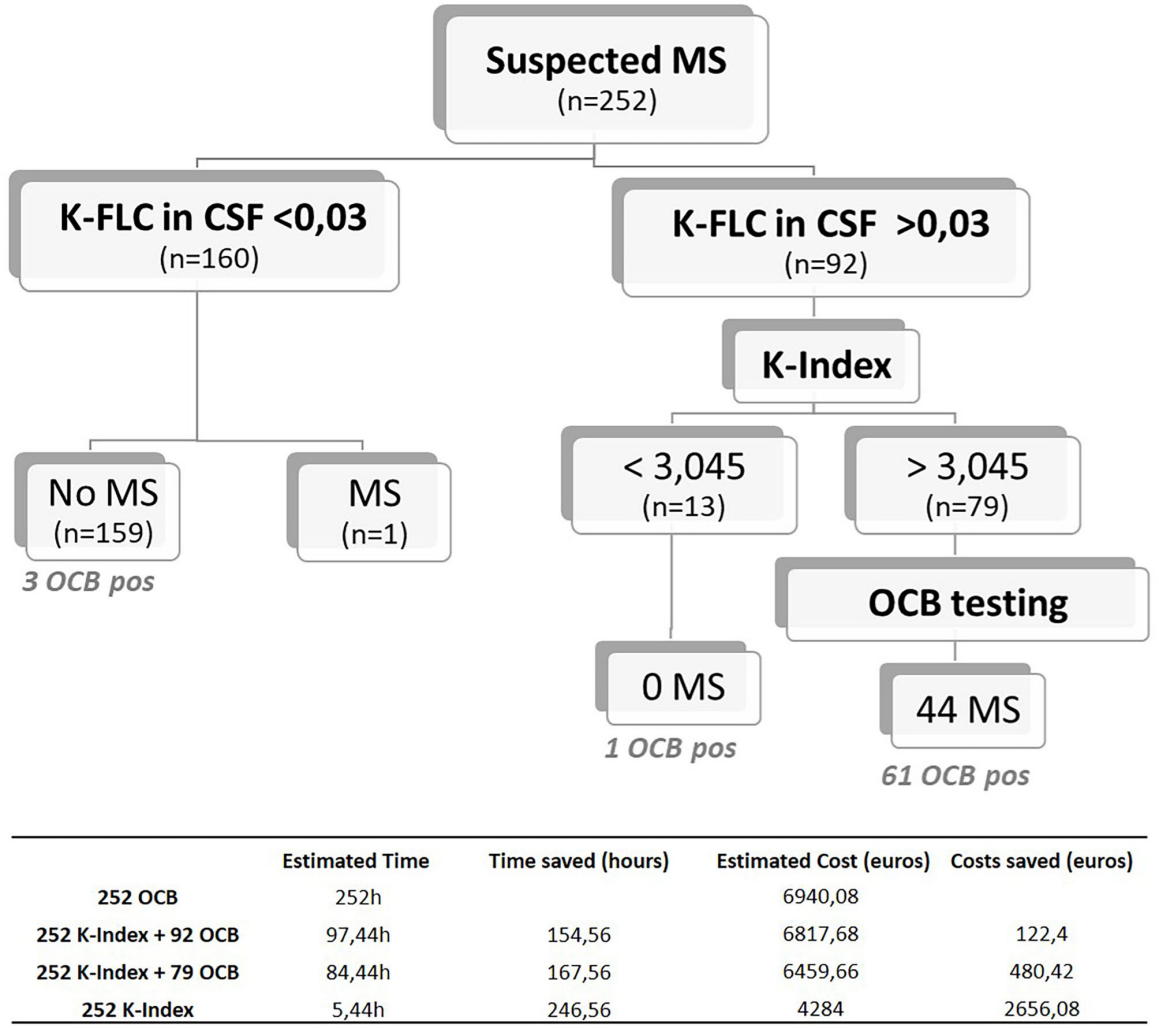
Proposed screening algorithm for patients with suspected MS, based on the K-FLC levels in CSF measured in an Optilite system, the K-Index 3.045 optimal threshold, and OCB testing. Estimated time and cost breakdown included on the table below. MS, multiple sclerosis; K-FLC, kappa free light chain; CSF, cerebrospinal fluid; K-Index, kappa index; OCB, oligoclonal band.

**Figure 5:**
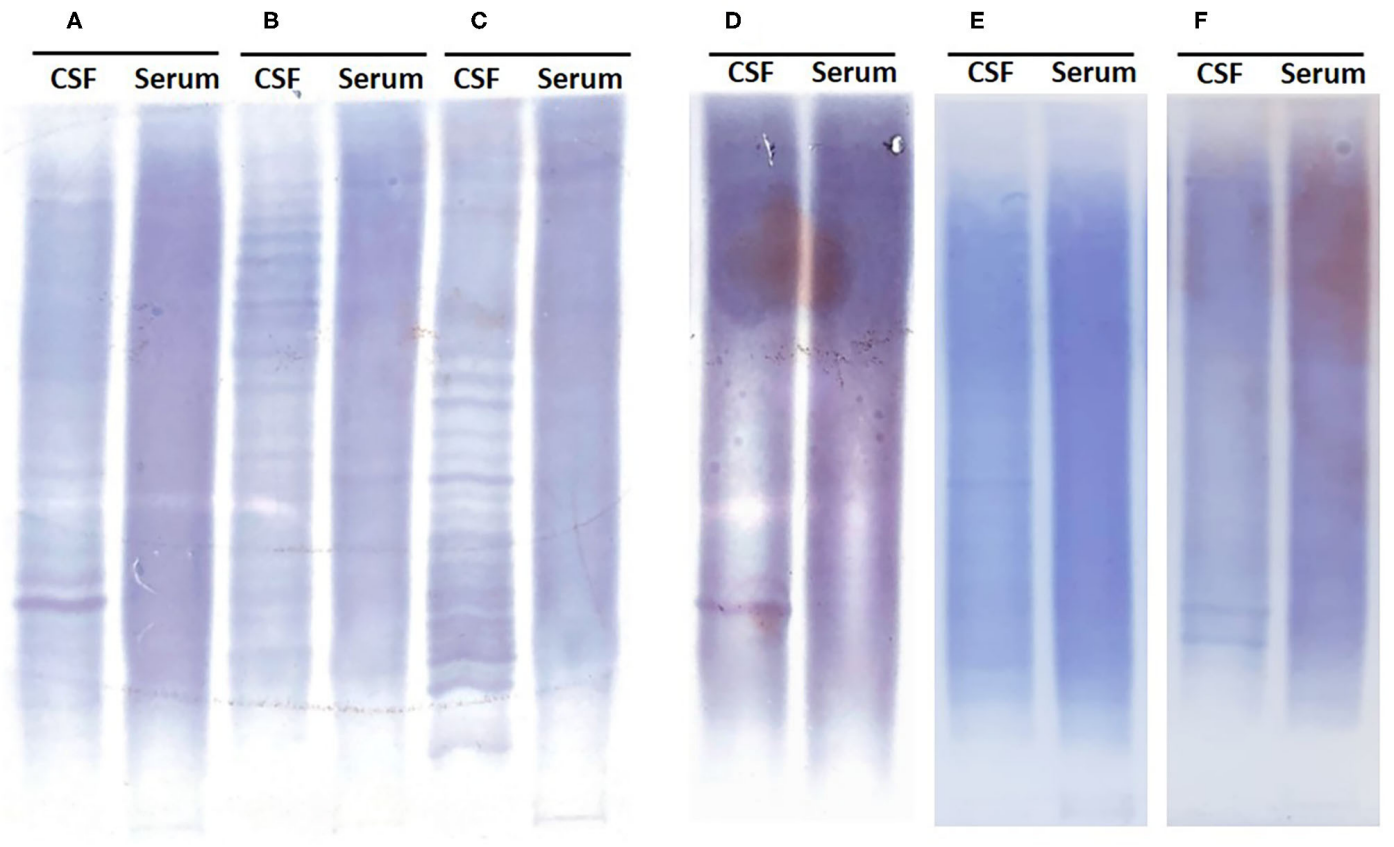
Representative high-resolution electrophoresis reflecting the difficulty on reporting OCB results in specific cases. **(A–C)** Easily identifiable bands in CSF not present in the serum sample. **(D)** Guillain–Barré syndrome with a K-Index of 39.98, one easy to distinguish and possibly a second OCB, making it difficult to report the OCB status. **(E)** MS with a K-Index of 39.29, faint bands on CSF impossible to confirm on serum due to strong background, reported negative OCB. **(F)** Recurrent left optic neuritis without MS, with K-FLC levels < 0.03 and two reported OCB. OCB, oligoclonal band; CSF, cerebrospinal fluid; K-Index, kappa index; MS, multiple sclerosis; K-FLC, kappa free light chain.

Based on the K-FLC absolute levels in CSF, K-Index, and OCB testing, a diagnostic algorithm was designed for screening suspected MS. With the presented population that includes all testing performed in our center in a period of 2 years, only one ptient with MS exhibited K-FLC levels in CSF below the detection limit of the method (K-FLC < 0.03 mg/dl). Because this patient did not show detectable OCB, which would be indicative of intrathecal immunoglobulin synthesis, we considered it justified to obviate OCB testing in patients with suspected MS showing K-FLC CSF levels <0.03 mg/dl, unless there is a strong clinical suspicion. If OCB testing had been reserved only for patients presenting K-FLC CSF levels higher than 0.03 mg/dl (OCB *n* = 92 samples, instead of 252), this approach would have reached a sensitivity similar to that of OCB alone (sensitivity = 0.8889) with a slightly higher specificity (0.9239) (*p* < 0.0001), a positive predictive value (PPV) of 72.73%, and, more interestingly, a negative predictive value (NPV) of 97.33%. Therefore, these results support the proposed strategy of reserving the OCB testing for cases with suspected MS presenting with K-FLC levels in CSF above 0.03 mg/dl. In our cohort, it would have meant a total saving of ~154.56 h of labor time and an estimated saving of 122.4 euros in reagent costs (Figure 4 and Table included). The time required for OCB validation is also of relevance since subjectivity is inherent to the technique. Figure 5 shows examples of samples that were difficult to interpret (D, E, and F) due to either faint bands or strong background that made it difficult to interpret, sometimes even requiring OCB to be reran, generating additional reagent cost and time.

According to our results, a second tier on the algorithm can be based on the K-Index value obtained, where OCB testing could be dispensable for patients with a K-Index < 3.045, since in our cohort no MS was diagnosed in this subgroup. When suspecting MS, testing K-FLC CSF levels (cutoff 0.03 mg/dl) followed by K-Index analysis (cutoff 3.045) achieves a maximum diagnostic sensitivity of 0.9778, with a specificity of 0.8629 (*p* < 0.0001) (PPV = 61.97%; NPV = 99.42%) (Figure 4). Reaching an NPV of almost 100% supports this strategy of reflexing OCB testing only if the K-Index is higher than 3.045, which would have spared ~68.7% (160 plus 13 out of 252 CSF samples evaluated) of all the OCB testing in our laboratory without losing any MS with intrathecal immunoglobulin synthesis. This combination of K-FLC levels, K-Index, and OCB techniques would save extra time (167.56 h for this cohort) and 480.42 euros in reagent costs, and we estimate that the final savings would be even higher if the specialized staff time required for OCB validation had been included in the cost estimation.

## Discussion

Routine CSF studies have been for a long time implemented in our laboratory for MS evaluation. OCB testing has been considered the gold standard for assessing intrathecal synthesis of immunoglobulin, aiding in the MS diagnosis as it has been revised in the 2017 McDonald criteria (7). In the present study, we propose a diagnostic algorithm to determine intrathecal synthesis of immunoglobulin, based on the determination of CSF free light chain kappa and K-Index prior to OCB testing.

From the laboratory point of view, there are practical benefits on the measure of K-FLC levels over testing for OCB, such as the complete automatization of the technique, the final objective quantitative result, and a lesser dependence on the expertise of the laboratory staff. Moreover, according to previously published data, K-Index achieves a comparable sensitivity and specificity to OCB for the diagnosis of MS (13, 14, 17, 18, 21, 22). The 3.045 K-Index cutoff here identified achieved a high sensitivity of 97.78% and a specificity of 86.29% for the MS diagnosis, therefore, reaching a higher sensitivity at the expense of a slightly inferior specificity when compared with the OCB results.

The diagnostic performance of this K-Index cutoff of 3.045 is in agreement with other previous published reports, such as Valencia-Vera et al. (13), who found a similar specificity of 85.66% for a close K-Index cutoff (2.91), albeit sensitivity in their cohort reached only 83.78%; and Agnello et al. (23), who also proposed a 2.9 cutoff to screen for MS with a sensitivity almost identical to that found in our present study (97.4%), although, at a lower specificity (64.7%), which may be explained by a smaller cohort and a higher proportion of MS patients included in that study. On the largest multicentric study done so far, Leurs et al. described a K-Index 6.6 cutoff that, in our population, achieved a slightly higher sensitivity and specificity (93.33 vs. 88% and 87.31 vs. 83%, respectively), once more validating its efficacy. This cutoff is also close to that defined on the Presslauer et al. report (K-Index cutoff = 5.9), which reached a sensitivity and a specificity of 96 and 86%, respectively, very close to the values here obtained with a 6.6 K-Index cutoff. Altogether, these results support the use of the K-FLC and K-Index as putative surrogate biomarkers for intrathecal immunoglobulin synthesis.

Results also showed that the cutoff could be geared toward a better sensitivity or a better specificity, since a higher K-Index cutoff of 12.58 achieved a specificity similar to the OCB (90.36 vs. 90.86%, respectively). However, the scope of this study was to optimize the screening protocol for suspected MS; thus, we favored sensitivity over specificity and choose the 3.045 K-Index cutoff. Importantly, in our cohort, we found no case with MS and positive OCB that would have been negative in our algorithm, supporting that the technical limit of detection of K-FLC in CSF in our platform is sufficiently low, not enough to affect the performance of the assay for the needed purpose. Moreover, Ferraro et al. (24) recently published a study of a population with suspected MS that presented no bands in OCB testing, where a K-Index of 5.8 identified about one-quarter of the OCB-negative MS patients, therefore, indicating a higher sensitivity over OCB. In our cohort, four out of five MS patients that were negative for OCB had a K-Index higher than 3.045, three of whom had K-Index markedly above 6.6, corroborating the high sensitivity for detecting intrathecal immunoglobulin synthesis. Notably, one MS patient showed no signs of intrathecally synthesized immunoglobulin by both negative OCB and undetectable K-FLC, a result that reminds that OCB is not pathognomonic of MS and that even with higher sensitive CSF-techniques, the MS diagnosis is complex and relies on a combination of clinical and MRI findings to support it.

The attribution of a close-to-zero residual value (in this case, an empirical 0.0001 mg/dl) to all samples presenting with K-FLC levels < 0.03 mg/dl impacts the group's K-Index median obtained. However, after exclusion of the 160 patients with K-FLC CSF levels <0.03 mg/dl from the analysis, the median K-Index obtained was still significantly different between MS and non-MS patients (*p* < 0.0001), with an increase in the K-Index median values particularly for the group of patients without MS. Likewise, a significant discriminatory capacity of the K-Index was also found by ROC curve analysis performed with these settings (AUC 0.8020; *p* < 0.0001; Supplementary Figure 1). Taking solely this cohort of patients with measurable K-FLC CSF levels >0.03 mg/dl, the sensitivity of OCB testing and of a 3.045 cutoff K-Index held high (0.9091 and 1.00, respectively), although, a substantial drop was observed as a result of excluding a significant number of cases from the control group of patients without an MS diagnosis (0.625 and 0.325, respectively).

The K-FLC and K-Index can also be explored for prognostic purposes such as the conversion of a CIS to MS, as proposed elsewhere (25, 26). However, in our center from 2018 to early 2020, there were only one patient diagnosed with CIS and two patients diagnosed with RIS, and all of them presented very high K-Index suggestive of a high risk of conversion to MS.

OCB is currently considered the gold standard of CSF studies for MS and is included in the 2017 McDonald criteria. Given the good sensitivity achieved and the easiness of their implementation, several studies support the K-FLC in CSF and the K-Index analysis to be considered in future criterion reviews as useful diagnostic tools and an adequate alternative to OCB. In this regard, the best decision cutoffs for this purpose would have to be defined. Specifically, for our laboratory, only about 17% of the CSF studies performed are finally diagnosed with MS. The application of the proposed screening algorithm, supported by a high NPV, would have prevented up to 173 (66%) of the 252 OCB studies from being performed, resulting in a more cost-efficient diagnostic protocol. Moreover, with the higher sensitivity found at an almost identical specificity, the replacement of OCB studies in the initial steps by the K-FLC CSF plus K-Index approach would offer a faster result for intrathecal immunoglobulin synthesis, allowing a quicker clinical management of patients if needed. In our practice, all clinical cases are deeply reviewed together by neurologists, radiologists, and biochemistry experts, and the samples are all stored at −80°C, so that OCB testing may be performed later in the event of a case with undetectable K-FLC levels but strong MS suspicion based on image or clinical findings. Still, visual scanning of the isoelectric focusing gels allow identification of different patterns of immunoglobulin distribution between serum and CSF samples that may be of interest for other specific clinical conditions and to establish clonality.

The presented study is limited by its monocentric nature and by the relatively small number of patients diagnosed with MS. However, it is the first time that the diagnostic value of K-FLC CSF is reported in a Canary Island population. The free light chain determinations were introduced in our hospital in routine since 2018, and this study collects all data from consecutive unselected patients referred for CSF analysis, therefore, representing a retrospective observational study on a complete and real-world cohort of patients for a period of more than 2 years. The number of MS diagnosis is within the expected for this period in our region, based on incidence rates (27). It is, to our knowledge, the first study validating the K-FLC CSF and K-Index as markers of intrathecal immunoglobulin synthesis in a Canary Island population. Unlike K-FLC, the lambda free light chains have been shown to be only moderately elevated in CSF samples from MS patients and a role in MS diagnosis is not proven; therefore, it has not been included in our routine analysis (6).

Despite being generally preserved in MS, it is important to correct for the putative impact of a compromised BCB on the diagnostic accuracy of the method. In our cohort, MS patients presented with a median albumin quotient of 0.004 (min. 0.0017; max. 0.012), with the highest albumin quotient found on a patient with suspected ependymoma (0.023). The K-Index linear function includes the albumin quotient, yet other reports have further evaluated non-linear functions to correct for possible alterations on the BCB by relating the kappa CSF/serum ratios to CSF/serum albumin ratio's dependent reference curves (17, 21, 28), obtaining excellent diagnostic accuracy but not proving to improve upon the easier-to-calculate K-Index (29–31). Moreover, we applied the recently defined hyperbolic function described by Reiber et al. (32) to estimate the K-FLC intrathecal fraction (IF) in our cohort of patients (data not shown), finding an almost absolute agreement with our algorithm results. Only one patient had a negative K-FLC fraction positively identified by our proposed algorithm. This MS patient had been diagnosed in 2003, and a new CSF analysis was requested during this study period, presenting 5–10 positive OCB, K-Index of 3.3, an albumin quotient of 3.99 (×10^−3^), and MRI criteria for dissemination in space. Compared with the 6.6 K-Index cutoff, three more cases had positive K-FLC IF, one of which had MS and negative OCB and another with OCB and no MS. Although, a benefit in terms of sensitivity and specificity for MS was not observed in our population, with cutoffs defined for screening purposes, further studies should explore the non-linear formulas regarding its correlation with the physiological state, and in other inflammatory diseases. Yet from a pragmatic point of view, the K-Index has shown excellent diagnostic characteristics, it is of global reach requiring simple calculations, and it is easy to report and interpret.

In summary, we propose an optimized protocol based on the initial use of automatized K-FLC in CSF and K-Index values to screen for intrathecal immunoglobulin synthesis and determine which samples will really benefit from performing OCB, ensuring excellent and cost-effective diagnostic performance based on a standardizable and globally available technique. Future validation of the screening algorithm, ideally by prospective multicentric and broader studies, is needed and assured.

## Data Availability Statement

The original contributions presented in the study are included in the article/Supplementary Material, further inquiries can be directed to the corresponding author/s.

## Ethics Statement

The studies involving human participants were reviewed and approved by ethical committee of the University Hospital of Nuestra Señora de Candelaria (CHUNSC 2020_110). Written informed consent for participation was not required for this study in accordance with the national legislation and the institutional requirements.

## Author Contributions

CS and SH contributed to conception and design of the study, collection of clinical and immunological data, statistical analysis, and manuscript writing. MC contributed with data collection and manuscript revision. MH and VM collected clinical and radiological data, respectively. All authors contributed to the article and approved the submitted version.

## Conflict of Interest

The authors declare that the research was conducted in the absence of any commercial or financial relationships that could be construed as a potential conflict of interest.

## Publisher's Note

All claims expressed in this article are solely those of the authors and do not necessarily represent those of their affiliated organizations, or those of the publisher, the editors and the reviewers. Any product that may be evaluated in this article, or claim that may be made by its manufacturer, is not guaranteed or endorsed by the publisher.

## Supplementary Material

Supplementary Figure 1 ROC curve and contingency analysis on the K-Index diagnostic performance for MS, after exclusion of cases presenting with K-FLC CSF levels below 0.03mg/dl.

Supplementary Figure 2 Mann-Whitney analysis of patients with a MS diagnosis vs. patients without MS, after exclusion of cases presenting with K-FLC CSF levels below 0.03mg/dl.

Supplementary Table 1 Case studies with inconclusive diagnosis where it was not possible to definitely rule out MS.

## References

[1] Wingerchuk DM Lucchinetti CF Noseworthy JH. Multiple sclerosis: current pathophysiological concepts. Lab Investig. (2001) 81:263–81. 10.1038/labinvest.3780235 11310820

[2] Comi G Filippi M Barkhof F Durelli L Edan G Fernández O. Effect of early interferon treatment on conversion to definite multiple sclerosis: a randomised study. Lancet. (2001) 357:1576–82. 10.1016/S0140-6736(00)04725-5 11377645

[3] Goodin DS Bates D. Review: treatment of early multiple sclerosis: the value of treatment initiation after a first clinical episode. Mult Scler J. (2009) 15:1175–82. 10.1177/1352458509107007 19737851

[4] Hickey WF. Migration of hematogenous cells through the blood-brain barrier and the initiation of CNS inflammation. Brain Pathol. (1991) 1:97–105. 10.1111/j.1750-3639.1991.tb00646.x 1669702

[5] Freedman MS Thompson EJ Deisenhammer F Giovannoni G Grimsley G Keir G. Recommended standard of cerebrospinal fluid analysis in the diagnosis of multiple sclerosis: a consensus statement. Arch Neurol. (2005) 62:865–70. 10.1001/archneur.62.6.865 15956157

[6] Presslauer S Milosavljevic D Brücke T Bayer P Hübl W. Elevated levels of kappa free light chains in CSF support the diagnosis of multiple sclerosis. J Neurol. (2008) 255:1508–14. 10.1007/s00415-008-0954-z 18685917

[7] Thompson AJ Banwell BL Barkhof F Carroll WM Coetzee T Comi G. Diagnosis of multiple sclerosis: 2017 revisions of the McDonald criteria. Lancet Neurol. (2018) 17:162–73. 10.1016/S1474-4422(17)30470-2 29275977

[8] Rudick RA Peter DR Bidlack JM Knutson DW. Multiple sclerosis: free light chains in cerebrospinal fluid. Neurology. (1985) 35:1443–9. 10.1212/WNL.35.10.1443 3929159

[9] Rudick RA Pallant A Bidlack JM Herndon RM. Free kappa light chains in multiple sclerosis spinal fluid. Ann Neurol. (1986) 20:63–9. 10.1002/ana.410200111 3090930

[10] Bracco F Gallo P Menna R Battistin L Tavolato B. Free light chains in the CSF in multiple sclerosis. J Neurol. (1987) 234:303–7. 10.1007/BF00314285 3112315

[11] DeCarli C Menegus MA Rudick RA. Free light chains in multiple sclerosis and infections of the CNS. Neurology. (1987) 37:1334–8. 10.1212/WNL.37.8.1334 3112610

[12] Senel M Tumani H Lauda F Presslauer S Mojib-Yezdani R Otto M. Cerebrospinal fluid immunoglobulin kappa light chain in clinically isolated syndrome and multiple sclerosis. PLoS ONE. (2014) 9:e88680. 10.1371/journal.pone.0088680 24695382 PMC3973621

[13] Valencia-Vera E Martinez-Escribano Garcia-Ripoll A Enguix A Abalos-Garcia C Segovia-Cuevas MJ. Application of κ free light chains in cerebrospinal fluid as a biomarker in multiple sclerosis diagnosis: development of a diagnosis algorithm. Clin Chem Lab Med. (2018) 56:609–13. 10.1515/cclm-2017-0285 29087953

[14] Leurs CE Twaalfhoven HAM Lissenberg-Witte BI van Pesch V Dujmovic I Drulovic J. Kappa free light chains is a valid tool in the diagnostics of MS: a large multicenter study. Mult Scler J. (2020) 26:912–23. 10.1177/1352458519845844 31066634 PMC7350201

[15] Desplat-Jégo S Feuillet L Pelletier J Bernard D Chérif AA Boucraut J. Quantification of immunoglobulin free light chains in cerebrospinal fluid by nephelometry. J Clin Immunol. (2005) 25:338–45. 10.1007/s10875-005-5371-9 16133990

[16] Duranti F Pieri M Centonze D Buttari F Bernardini S Dessi M. Determination of κFLC and κ index in cerebrospinal fluid: a valid alternative to assess intrathecal immunoglobulin synthesis. J Neuroimmunol. (2013) 263:116–20. 10.1016/j.jneuroim.2013.07.006 23916392

[17] Presslauer S Milosavljevic D Huebl W Aboulenein-Djamshidian F Krugluger W Deisenhammer F. Validation of kappa free light chains as a diagnostic biomarker in multiple sclerosis and clinically isolated syndrome: a multicenter study. Mult Scler. (2016) 22:502–10. 10.1177/1352458515594044 26199348

[18] Crespi I Sulas MG Mora R Naldi P Vecchio D Comi C. Combined use of kappa free light chain index and isoelectrofocusing of cerebro-spinal fluid in diagnosing multiple sclerosis: performances and costs. Clin Lab. (2017) 63:551–9. 10.7754/Clin.Lab.2016.160930 28271695

[19] Süße M Hannich M Petersmann A Zylla S Pietzner M Nauck M. Kappa free light chains in cerebrospinal fluid to identify patients with oligoclonal bands. Eur J Neurol. (2018) 25:1134–9. 10.1111/ene.13667 29683546

[20] Sanz Díaz CT Pérez Hernández LM Hernández Pérez MA González-Plata M. Implementación y revisión del protocolo para el diagnóstico de la esclerosis múltiple en nuestro hospital. An Clin. (2006) 31:101–6.

[21] Presslauer S Milosavljevic D Huebl W Parigger S Schneider-Koch G Bruecke T. Kappa free light chains: diagnostic and prognostic relevance in MS and CIS. PLoS ONE. (2014) 9:e89945. 10.1371/journal.pone.0089945 24651567 PMC3940961

[22] Gurtner KM Shosha E Bryant SC Andreguetto BD Murray DL Pittock SJ. CSF free light chain identification of demyelinating disease: comparison with oligoclonal banding and other CSF indexes. Clin Chem Lab Med. (2018) 56:1071–80. 10.1515/cclm-2017-0901 29455184

[23] Agnello L Lo Sasso B Salemi G Altavilla P Pappalardo EM Caldarella R. Clinical use of κ free light chains index as a screening test for multiple sclerosis. Lab Med. (2020) 51:402–7. 10.1093/labmed/lmz073 31943078

[24] Ferraro D Trovati A Bedin R Natali P Franciotta D Santangelo M. Cerebrospinal fluid kappa and lambda free light chains in oligoclonal band-negative patients with suspected multiple sclerosis. Eur J Neurol. (2020) 27:461–7. 10.1111/ene.14121 31710409

[25] Villar LM Espiño M Costa-Frossard L Muriel A Jiménez J Álvarez-Cermeño JC. High levels of cerebrospinal fluid free kappa chains predict conversion to multiple sclerosis. Clin Chim Acta. (2012) 413:1813–6. 10.1016/j.cca.2012.07.007 22814197

[26] Menéndez-Valladares P García-Sánchez M Cuadri Benítez P Lucas M Adorna Martínez M Carranco Galán V. Free kappa light chains in cerebrospinal fluid as a biomarker to assess risk conversion to multiple sclerosis. Mult Scler J - Exp Transl Clin. (2015) 1:1–9. 10.1177/2055217315620935 28607709 PMC5433434

[27] Aladro Y Alemany MJ Pérez-Vieitez MC Amela R Conde M Reyes MP. Prevalence and incidence of multiple sclerosis in Las Palmas, Canary Islands, Spain. Neuroepidemiology. (2005) 24:70–5 10.1159/000081052 15459512

[28] Senel M Mojib-Yezdani F Braisch U Bachhuber F Lewerenz J Ludolph AC. CSF free light chains as a marker of intrathecal immunoglobulin synthesis in multiple sclerosis: a blood-CSF barrier related evaluation in a large cohort. Front Immunol. (2019) 10:641. 10.3389/fimmu.2019.00641 30984199 PMC6449445

[29] Hegen H Walde J Milosavljevic D Aboulenein-Djamshidian F Senel M Tumani H. Free light chains in the cerebrospinal fluid. Comparison of different methods to determine intrathecal synthesis. Clin Chem Lab Med. (2019) 57:1574–86. 10.1515/cclm-2018-1300 31112501

[30] Puthenparampil M Altinier S Stropparo E Zywicki S Poggiali D Cazzola C. Intrathecal K free light chain synthesis in multiple sclerosis at clinical onset associates with local IgG production and improves the diagnostic value of cerebrospinal fluid examination. Mult Scler Relat Disord. (2018) 25:241–5. 10.1016/j.msard.2018.08.002 30130707

[31] Emersic A Anadolli V Krsnik M Rot U. Intrathecal immunoglobulin synthesis: the potential value of an adjunct test. Clin Chim Acta. (2019) 489:109–16. 10.1016/j.cca.2018.12.006 30529605

[32] Reiber H Zeman D Kušnierov P Mundwiler E Bernasconi L. Diagnostic relevance of free light chains in cerebrospinal fluid - the hyperbolic reference range for reliable data interpretation in quotient diagrams. Clin Chim Acta. (2019) 497:153–62. 10.1016/j.cca.2019.07.027 31351929

