# Supplementary figures and images for: Evaluation of Kappa Index as a Tool in the Diagnosis of Multiple Sclerosis: Implementation in Routine Screening Procedure
Source: Front Neurol. 2021 Aug 11;12:676527. doi: 10.3389/fneur.2021.676527 (PMC8386692; doi:10.3389/fneur.2021.676527)

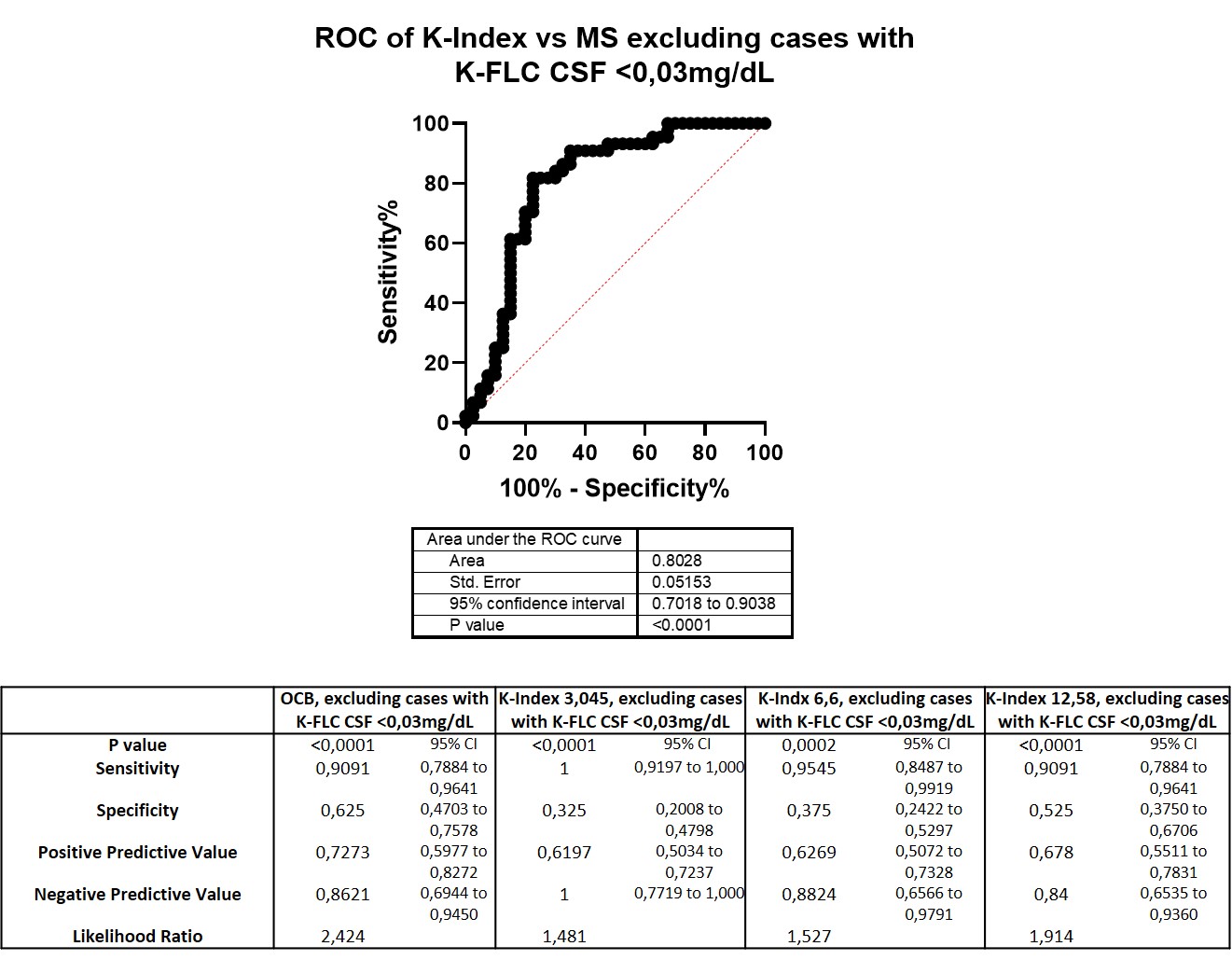

Supplement: Supplementary Figure 1 — ROC curve and contingency analysis on the K-Index diagnostic performance for MS, after exclusion of cases presenting with K-FLC CSF levels below 0.03mg/dl. [file Image_1.JPEG]

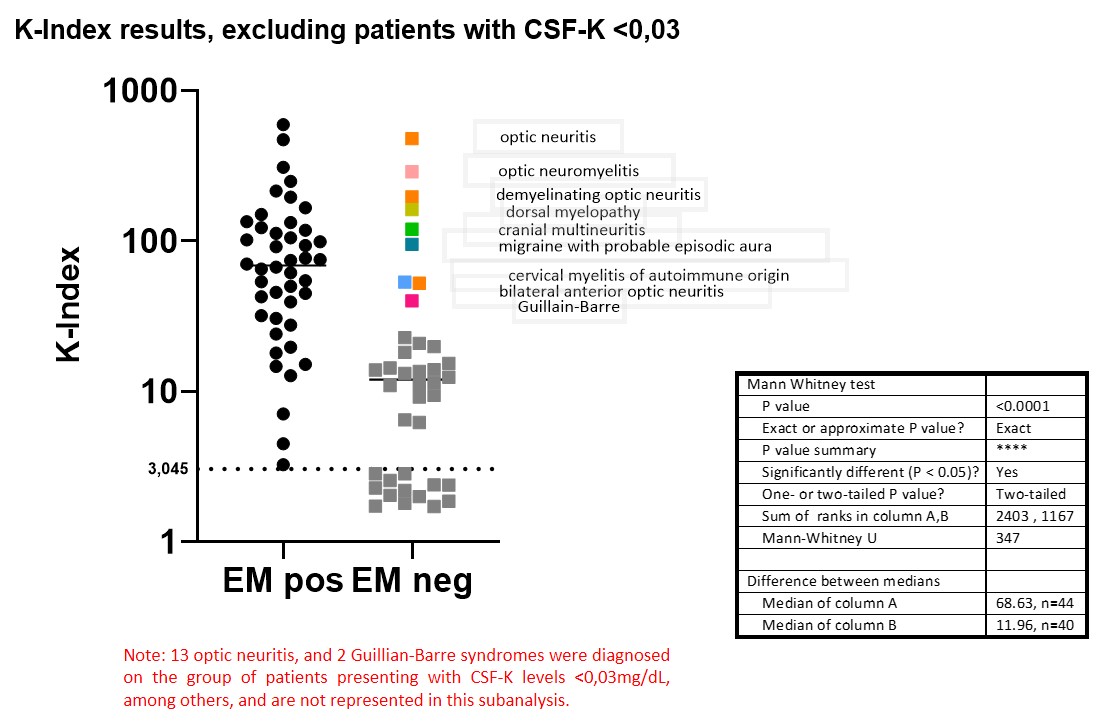

Supplement: Supplementary Figure 2 — Mann-Whitney analysis of patients with a MS diagnosis vs. patients without MS, after exclusion of cases presenting with K-FLC CSF levels below 0.03mg/dl. [file Image_2.JPEG]
