# Supplementary material for: Evaluation of Kappa Index as a Tool in the Diagnosis of Multiple Sclerosis: Implementation in Routine Screening Procedure
Source: Front Neurol. 2021 Aug 11;12:676527. doi: 10.3389/fneur.2021.676527 (PMC8386692; doi:10.3389/fneur.2021.676527)
Supplement: Supplementary Table 1 — Case studies with inconclusive diagnosis where it was not possible to definitely rule out MS. [file Table_1.DOCX]

**Supplementary table 1:** Case studies with inconclusive diagnosis where it was not possible to definitely rule out MS.

| **K-Index** | **OCB** | **n (bands)** | **MRI** | **MRI details** | **Diagnosis** |
| --- | --- | --- | --- | --- | --- |
| 0,031 | neg. | 0 | inconclusive | High-signal lesion in T2-weighted sequences and left thalamus-midbrain FLAIR, with elongated morphology, extending caudally from the thalamus. | Possible Behçet, NMO, venous vasculitis |
|  |  |  |  |  |  |
| 0,02 | neg. | 0 | pos. | Multifocal leukopathy that meets criteria for MS’ dissemination in space. | Very doubtful symptoms, probable PPMS? Rapid progression with MII amyotrophy. PNP data without VRF; PNP data |
|  |  |  |  |  |  |
| 237,24 | pos. | 10-15 | inconclusive | Isolated subcentimetric left bulbo-pontine lesion, anterior to the nucleus of the left sixth nerve, with nonspecific behaviour, without evidence of other associated lesions. | Probable demyelinating disease |
|  |  |  |  |  |  |
| 372,33 | pos. | 15-20 | inconclusive | Lesion with demyelinating characteristics at the D10 level. Brain MRI: nonspecific small lesions. | Dorsal myelopathy of suspected inflammatory etiology |
|  |  |  |  |  |  |
| 413 | pos. | 15-20 | inconclusive | 2 periventricular lesions with a demyelinating appearance of the brain, under study | Pompe disease, inflammatory CSF + clinical episodes compatible with outbreaks. Spatial dissemination criteria missing for the diagnosis of multiple sclerosis |
|  |  |  |  |  |  |
| 98,56 | pos. | 10-15 | neg. | Multiple areas of signal alteration affecting subcortical regions, due to well-defined lesions, without significant expression in diffusion sequence or gradient of probable microangiopathic aetiology. | Polyneuropathy and Sjögren's syndrome |
|  |  |  |  |  |  |
| 8,49 | neg. | 0 | inconclusive | Except for the lesion described, of probable demyelinating aetiology, the rest of the findings suggest small-vessel ischemic leukopathy. | Severe right optic neuropathy under study |
